# Adherence to acne treatment guidelines in the military environment - a descriptive, serial cross-sectional study

**DOI:** 10.1186/s40779-015-0063-0

**Published:** 2015-12-23

**Authors:** Chen Fleischmann, Lior Cohen, Elimelech Adams, Michael Hartal

**Affiliations:** Israel Defense Forces Medical Corps, IDF, P.O. Box 02149, Te Hashomer, Israel; Department of Military Medicine, Faculty of Medicine, Hebrew University, Jerusalem, Israel

**Keywords:** Acne, Treatment, Medications, Guidelines, Military

## Abstract

**Background:**

Acne vulgaris, a common skin disease, affects up to 80 % of the population. Moderate to severe acne requires treatment with a combination of topical and oral drugs such as antibiotics, hormones and retinoids. Retinoids have many contraindications and adverse effects requiring close monitoring. The study’s objectives were to describe prescribing trends in acne medication over time in a military setting, and assess physician adherence to guidelines for acne treatment, including drug precautions, clinical monitoring, and treatment progression.

**Methods:**

We conducted a descriptive, serial cross-sectional study of acne drugs prescribed in the Israel Defense Forces (IDF) in the years 2002–2007, analyzing the classes of drugs prescribed and patient characteristics. In addition, the clinical quality of the medical encounter was assessed by examining physician adherence to IDF guidelines.

**Results:**

Between 2002 and 2007, 64,281 patients were treated for acne. Treatment courses generally persisted for 1–2 months. Over 70 % of female patients receiving oral retinoids were not concomitantly receiving oral contraceptives.

**Conclusion:**

This study provides a unique perspective of acne treatment in a military setting, overall displaying good adherence to general guidelines. The common prescription of oral retinoids to young females without concomitant contraception is alarming.

## Background

Acne vulgaris is a common inflammatory skin disease with a complex and multifactorial pathogenesis. Increased sebum secretion leads to seborrhea and comedogenesis, while bacterial infection of the comedo, usually by *Propionibacterium acnes*, causes inflammation leading to papules, pustules and nodules [1–3]. Acne commonly appears on the face, but may also be found on the upper chest, shoulders and back [2]. It can result in severe cosmetic blemishing and can be emotionally distressing to affected individuals, especially to adolescents and young adults [4].

Acne vulgaris affects over 80 % of the population at some stage in life, especially young adults. Despite the high disease prevalence, only about 20–30 % of patients seek medical treatment [1, 5–7]. Gender distribution is roughly equal [1, 8, 9] although during adolescence males are more affected than females [8, 10]. Acne can last several years and normally resolves by the mid-twenties. In recent years, a gradual increase in the age of onset has been observed and in over 8 % of patients the onset is after the age of 25, more commonly in women [11].

In the treatment of mild acne, proper hygiene with antiseptic non-abrasive soap is recommended, as well as a well-balanced diet [2]. If the problem fails to resolve spontaneously, topical treatment is usually recommended, including antibiotics such as clindamycin, erythromycin and tetracycline, retinoids including tretinoin, isotretinoin and the tretinoin-like drug adapalene, salicylic acid, azelaic acid and benzoyl peroxide as mono or combination therapy [1]. In recent years, antibiotic-resistant bacterial strains seem to be developing, and acne is somewhat less responsive to topical anti-bacterial treatment with erythromycin [12].

Oral contraceptive (OC) treatment may also be considered as a treatment option for mild acne in women. In young women, acne is responsive to OC treatment because it reduces the androgenic effects on increased sebum secretion [13, 14].

In moderate acne, a second line of treatment is required with orally administered systemic antibiotics, the most common of which is minocyclin, with other tetracyclines such as doxycycline, tetracycline and oxytetracycline in clinical use [1, 15]. Oral contraceptives are effective in women with moderate acne, and all oral therapies may be combined with topical agents for increased effect.

In severe acne, the third and definitive line of treatment is oral isotretinoin (OI) which is a synthetic form of vitamin A. It significantly reduces size and sebum production of the sebaceous glands, normalizes follicular keratinization and prevents the development of micro-comedones and comedones, indirectly inhibits P. acnes growth by changing the follicular milieu, and exerts direct anti-inflammatory activity. Results of OI treatment may be apparent only after a few weeks, but a full course of treatment can take up to 6 months. OI is contraindicated in pregnancy because it is highly teratogenic, and ruling out pregnancy is required before commencement of therapy in females [16]. OIs are associated with several side effects which require close laboratory and clinical monitoring [2, 5–7, 17, 18].

### Acne in the military setting

Military service is mandatory in Israel, and the young adults that serve in the Israel Defense Forces (IDF) represent the majority of males aged 18–21 and females aged 18–20. The remainder of the military population is comprised of career and reserve soldiers aged 21 to 45. During military service, young adults, who are naturally predisposed to acne vulgaris, are exposed to high levels of physical and psychological stress, in an environment often characterized by poor sanitary conditions.

Military organizations worldwide recognize acne as a significant healthcare issue affecting the daily lives of soldiers: Acne was reported as one of the top 5 skin conditions affecting military personnel in the Singapore military [19]. Between 1996 and 1997 16 % of patients (military and civilian personnel) treated in an Oslo military clinic were suffering from skin disease, 10 % of whom were suffering from acne vulgaris [20]. Researchers recommended the appointment of a dermatologist to the military medical team. More recently, acne was found to be among the three most common skin problems (35.6 %) in the Korean military, along with tinea pedis (15.2 %) and atopic dermatitis (5.1 %) [21].

In the IDF, acne is treated by general practitioners in primary care clinics, and by medical specialists, such as dermatologists, family practitioners and gynecologists, in secondary care clinics. The clinical guidelines for acne treatment published by the IDF Medical Corps call for treatment of mild acne with topical preparations, and consideration of OC treatment in women. For moderate acne, combination therapy with oral antibiotics and local treatment is recommended. For severe acne, OI is the drug of choice. Since contraindications for OI treatment include pregnancy and lactation, before commencing therapy the prescribing physician must ensure, through reliable laboratory tests, that the female patient is not pregnant. The physician is required to explain to the patient that reliable means of birth control must be in use for at least one month prior to treatment, during the entire course of the treatment, and for one month following cessation of treatment. The patient is required to sign a statement acknowledging the drug’s teratogenic nature and agreeing to take birth control measures for the duration of treatment, as well as agreeing to inform the treating physician in case of pregnancy. This statement is filed in the patient’s medical record. The concomitant use of OI and tetracycline or its derivatives is strongly contraindicated because of the risk of developing benign intracranial hypertension (pseudotumor cerebri). Also contraindicated are psychiatric reports of the patient having developed depression and/or suicidal tendencies in the past or present. Prior to commencement of and periodically throughout treatment, all patients must undergo baseline blood tests, including complete blood count, liver functions, blood lipids (cholesterol, HDL, LDL, triglycerides) and CPK levels, and daily dosages are adjusted accordingly. Accepted dosage is 0.5–1.0 mg/(kg•d) in two divided doses, over several months. The maximum daily dose should not exceed 120 mg.

Medical care in the IDF is provided to soldiers free of charge. Since no monetary barriers are in place, patients are likely motivated to seek treatment for medical conditions that might otherwise be considered minor or expensive, and we can expect to find within the IDF a large group of patients under medical treatment for acne. However, access to care may be limited for certain sub-groups, especially those serving under remote field conditions, leaving some lesser complaints untreated.

We conducted a descriptive, serial cross-sectional study of acne drugs prescribed in the IDF during the years 2002–2007. Our aim was to analyze the classes of drugs prescribed and patient characteristics, and to assess the clinical quality of the medical encounter by examining physician adherence to mandatory IDF clinical guidelines.

## Methods

### Data

The study database was constructed by combining records from two existing computerized databases: the computerized patient record (CPR), a comprehensive medical file resource, and the IDF pharmacy management program. The data were assembled and analyzed using SAS® software, version 9.2. The study was approved by the IDF Institutional Review Board in accordance with the Declaration of Helsinki. The database was de-identified and all information was analyzed while maintaining patient anonymity.

Doctor patient encounters between the years 2002 and 2007 were searched for the diagnosis of “acne” (ICD9 code 706.1X). Since this was the only option available to the examining physicians to code acne related encounters, this represents a high specificity and high sensitivity diagnosis. The IDF pharmacy management program was queried for all acne medications as defined in Appendix and cross referenced with the patient records arising from the CPR database, thus creating a record of acne therapy composed of prescribing and dispensing encounters for the same patients. For each patient with a diagnosis of acne, the first date of diagnosis in the CPR system was searched. For every initial diagnosis date located, the drug prescribing (CPR data) and dispensing (pharmacy data) dates were compared with the initial diagnosis date. Patients were included in the database only if the diagnosis preceded the prescribing or dispensing of acne medication. In order to avoid bias of medications prescribed to treat other conditions, we then filtered the results and identified distinct acne encounters limited solely to 1st, 2nd or 3rd line acne medications. All distinct acne encounters were filtered according to encounter year and type.

A treatment course was defined as beginning on the date of the first acne encounter with a specific group of acne medication and lasting until 30 days following the last date of prescribing or dispensing a medication of the same group. If a medication of the same group was prescribed or dispensed within 45 days of the previous date of prescription or dispensing of a medication of the same group, it was considered within the course of treatment.

Individual physicians’ clinical experience with acne treatment at the time of the encounter was obtained by counting the number of acne encounters recorded during the preceding 3 months and categorizing them into low (0–20 %), medium (20–30 %) and high (≥30 %) experience. Key IDF clinical guideline recommendations for the treatment of acne and their operational methods of assessment in the current study are presented in Table 1. Adherence to guidelines was evaluated by the appropriateness of treatment sequence and by the documented prescription of OC’s to female OI recipients. All treatments were referred to by the year of their initiation.

**Table 1:** Key IDF clinical guideline recommendations for the treatment of acne and their operational methods of assessment

Population	IDF guideline recommendation	Assessment in study
All acne patients	Assign treatment according to acne severity	Not assessed in study: could not be evaluated by computerized query (Disease severity not stated within diagnosis field)
All acne patients	Progress gradually between treatment lines	Correctness of treatment progression assessed for treatment courses of distinct acne medications (see Table 4)
Patients treated with oral retinoids	Rule out psychiatric diagnoses prior to treatment initiation, and refer to psychiatrist if necessary	Not assessed in study: information not available in computerized patient records
Patients treated with oral retinoids	Monitor laboratory test values (liver enzymes, plasma lipid profile) before and during treatment.	Not assessed in study: computerized laboratory test records were not available for cross analysis between databases.
Patients treated with oral retinoids	Prescribe supportive care if necessary.	Concomitant prescribing or dispensing of supportive care medications was assessed (see Table 5).
Patients treated with oral retinoids	Restrict patient participation in strenuous physical activity by temporarily adjusting medical profile.	Not assessed in study: documentation of temporary change in medical profile was not available after reinstatement of permanent profile.
Female patients treated with oral retinoids (teratogenic)	- Rule out pregnancy (laboratory test).	Documented concomitant prescribing or dispensing of oral contraceptives to female patients receiving OI’s.
	- Inform the patient of potential hazards and explain about required birth control measures.	
	- Have the patient sign a statement and file in their medical record (see background).	

### Analysis

All descriptive data of the patients, physicians and encounters were stratified by year. Continuous care was then analyzed in the form of treatment courses. Some missing information between the years 2002 and 2003, which we surmised to arise from the incomplete process of CPR software assimilation, led us to re–define and re-analyze the continuous elements of the information which could not be stratified annually, so that only the years with comprehensive data from all sources were included in the analysis. The non-stratified analyses therefore relate to the years 2004–2007. The lack of grading options in the CPR regarding acne severity prevented a stratified analysis of appropriateness of treatment. Therefore, we analyzed the treatment sequence according to medication type (1st, 2nd, 3rd line, OC’s, non-specific acne treatment, supportive care), as shown in Appendix. We also assessed the logical progression of treatment, from 1st to 2nd and 3rd lines. The assessment of physician monitoring of OI therapy included the prescribing of oral contraceptives in female patients prior to or during therapy, restricting the patients’ participation in strenuous physical activity and prescribing concomitant supportive care.

## Results

Over the study period, we identified 159,316 encounters with acne recorded as the diagnosis, representing 64,281 individual patients. Collectively, these patients were prescribed acne medications on 225,015 occasions. Computerized pharmacy data were available from 2004, allowing us to query dispensing encounters from this point on. During this 4 year period 50,942 patients received acne medication on 179,540 individual occasions yielding a rate of 3.52 prescriptions filled per patient. For acne patients, acne medications represented 41.7 % of all prescriptions written (Table 2).

**Table 2:** Patients, physician visits and prescription volumes, by study period (*n*)

Description	2002–2007	2004–2007
CPR encounters with acne diagnosis	159,316	129,672
Number of patients treated for acne	64,281	50,942
Prescribing encounters	225,015	193,313
All drug dispensing encounters	N/A	430,851
Acne drug dispensing encounters	N/A	179,540

N/A: not available

Patient characteristics are shown in Table 3. As expected in a conscript army, the vast majority of patients were aged 18–21, and nearly two thirds of patients (63.6 %) were male. Over 90 % of both males and females had a high school education. Nearly 80 % of patients were born in Israel, and their ethnic backgrounds were varied.

**Table 3:** Patient characteristics, by sex

Variable		Number	% of total	% male	% female
Patients		64,179	100.0	63.6	36.4
Age (year)					
	≤21	59,928	93.4	91.7	96.3
	22–28	3,656	5.7	7.3	2.9
	≥29	595	0.9	1.0	0.8
Education					
	Elementary	63	0.1	0.1	0.0
	High school	59,578	92.8	90.5	96.9
	Post-high school	4,415	6.9	9.1	2.9
	Unknown	123	0.2	0.2	0.2
Ethnicity					
	Israel	4,853	7.6	8.4	6.1
	Asia + Africa + Ethiopia	30,215	47.1	45.2	50.3
	West	15,329	23.9	24.6	22.7
	Former Soviet Union	8,187	12.8	13.3	11.9
	Unknown	5,595	8.7	8.6	9.0
Country of birth					
	Israel	50,726	79.0	78.4	80.1
	Asia, Africa	1,298	2.0	2.4	1.5
	West	1,861	2.9	3.1	2.6
	Former Soviet Union	6,776	10.6	10.8	10.1
	Unknown	3,518	5.5	5.3	5.8

Trends in patient volume over time are shown in Table 4. While the number of female patients increased gradually and remained stable form 2004 to 2007, the number of male patients peaked in 2005 and decreased through 2007. This pattern in male soldiers likely represents, at least in part, technical limitations of the data set in later years.

**Table 4:** Correctness of treatment sequence

Year of diagnosis	Patients (*n*)			Treatments (*n*)		
	Male	Female	Total	Incorrect	Correct	Total
2002	4,991	2,551	7,542	293	4,141	4,434
2003	6,651	3,071	9,722	423	6,115	6,538
2004	7,967	4,235	12,202	695	8,223	8,918
2005	8,905	4,451	13,356	677	9,054	9,731
2006	7,560	4,654	12,214	522	8,479	9,001
2007	4,727	4,416	9,143	204	6,274	6,478
Total	64,179	40,801	23,378	2814	42,286	45,100

The following treatment combinations and progressions were considered correct: 1st line therapy before or in combination with 2nd or 3rd line therapy; and 2nd before 3rd line therapy. The following were considered incorrect: 2nd before 1st line and 3rd before or in combination with 2nd line therapy

Slightly more than half of the encounters yielded a prescribed medication (56 %), and most were performed in primary care facilities (70 %). When prescribed, the treatment of choice was mainly 1st line monotherapy, followed by 2nd line, 3rd line and OC’s, with other non-distinct acne medications being the least chosen therapy. Supportive care was prescribed in 678 encounters (Table 5).

**Table 5:** Acne treatments by type, 2004–2007

Treatment	Number	Mono therapy	Combination therapy
1st line (topical)	54,999	43,325	11,674
2nd line (oral antibiotics)	26,645	15,878	10,767
3rd line (oral retinoid)	11,602	10,815	787
Oral contraceptives	3,347	3,003	344
Non-distinct acne medication	892	330	562
Supportive care	678	53	625

The majority of treating physicians (76 %) had limited medical expertise, and the majority of encounters were held in primary care facilities (97 %), suggesting that most of the acne diagnosis and care in the IDF is performed in primary care unit clinics by physicians less experienced with acne treatment. Treatment in secondary care facilities was relatively uncommon (2.7 % of all encounters), but was often given by more experienced physicians.

**Fig. 1:**
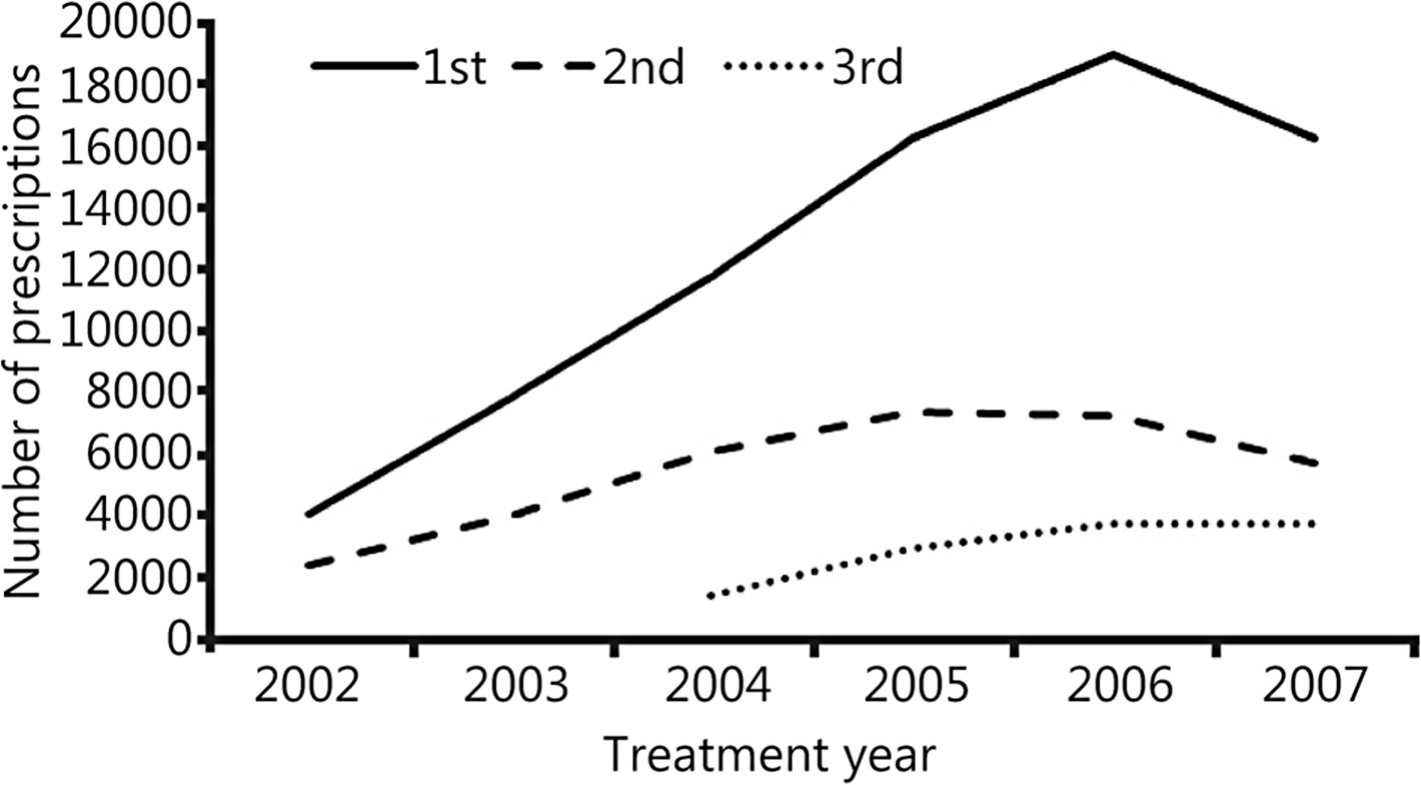
Trends in acne prescriptions over time, by line of therapy

Drug prescriptions peaked during 2006 at 28,491 prescriptions. Figure 1 shows trends over time in prescription volume for 1st, 2nd and 3rd lines of therapy. The use of 1st line therapy increased more than fourfold between 2002 and 2006, peaking at just under 19,000 prescriptions annually. Between 2002 and 2005, 2nd line drug prescriptions increased three fold, from 2,400 to 7,400 annually. While absolute numbers of 3rd line therapies were substantially lower, they more than doubled in annual volume between 2004 and 2006.

Therapy courses mostly persisted for no longer than 1–2 months. As expected, the majority of long-term treatments (spanning several months) were those of 3rd line therapies, however, most patients did not continue to take the full course of therapy (4–6 months) as commonly recommended. 4,234 correct treatment courses displayed progress from 1st to 2nd line therapy, representing 10 % of correct treatment courses, and 38 % of correct 2nd line combination therapy courses. 1,465 correct treatment courses displayed progress from 2nd to 3rd line therapy, representing 3.5 % of correct treatment courses, and 51.5 % of correct 3rd line combination therapy courses. Incorrect progression between drug lines represented less than 7 % of all treatments.

We examined all OI treatment courses in female patients for concomitant oral contraceptives, including treatment initiated 30 days before treatment commencement.

An alarming finding was that more than 70 % of female patients prescribed 3rd line therapy did not receive oral contraceptives during the course of the acne treatment or temporally adjacent to it.

## Discussion

Acne is a highly prevalent medical condition in the military population. Although not life-threatening, it does have some severe social and psychological ramifications. There may be additional operational impact, since the side effects of some acne medications include increased photosensitivity and additional symptoms which may be exacerbated among soldiers training and operating in heightened physical and mental stress and under extreme environmental conditions.

In this community setting of adolescents and young adults in the military, the majority of acne related patient-physician encounters ended with a prescribed medication. Most treatment courses were short, and most patients taking 3rd line medications failed to complete the full course of treatment. Incorrect progression between drug lines was rare.

The vast majority of female patients prescribed 3rd line therapy did not receive concomitant oral contraceptives.

The availability of computerized data on both clinical encounters and pharmacy dispensing provided a unique opportunity to describe the characteristics of acne treatment in a population of adolescents and young adults. The majority of the study population was male, which is not surprising in a military environment 44 % of patient encounters did not end in any drug prescription. It should be noted, however, that these data do not represent individual patients, but rather the encounters these patients underwent. It is possible that some of these encounters represent follow-up sessions performed by treating physicians, after having provided the patient with multiple prescriptions on a previous encounter. Another explanation could be referral of patients by unit physicians to secondary care facilities. This information can be of use as a benchmark by medical organizations providing volume of patient encounters. Trends in medication prescription increased over the study period, while the patient population remained stable. This may indicate increased patient awareness and demand for medications, increased physician prescribing habits or both. Our data indicate that the vast majority of acne diagnosis and care in the IDF is performed in primary care clinics by physicians less experienced with acne treatment. This observation underscores the importance of having clear and precise clinical guidelines in place to ensure a uniform standard of treatment. More than half of 3rd line treatment courses ended after 2 to 3 months, whereas treatment guidelines call for 4–6 months of continuous therapy in order to achieve maximal benefit in most patients. This finding may represent insufficient therapy due to poor patient compliance among soldiers in the military setting, or less than optimal treatment practices by physicians. However, this may in some measure indicate the high patient turnover in the military environment due to cyclic recruitment and discharge cycles of conscripted military personnel. Limited accessibility of medical treatment may also be a contributing factor. There may also be additional explanations for this finding such as disrupted treatment courses among soldiers serving far away from medical facilities where the medication could be dispensed, or low treatment compliance due to side effects.

The assessment of treatment progression demonstrated that the vast majority of treatments were chronologically correct. This finding is encouraging. However, it is noteworthy that at the time of this study, it was not possible to define the severity of the diagnosis, so that we were unable to examine the allocation of treatment according to the grade of acne.

The finding that oral contraceptives were infrequently used by female patients taking OI’s is concerning. It is possible that some female patients were, in fact, taking OC’s purchased outside of the military. Alternatively, this finding may represent a high rate of sexual abstinence in our study population. However, the possibility of sexual activity and unintentional pregnancy together with the teratogenic potential of OI should encourage physicians to prescribe, and patients to take, oral contraceptives during OI treatment. This issue should be stressed among prescribing physicians. It is also possible that treating physicians found the signing of a statement by the patient to be sufficient, since prescribing oral contraceptives was not an absolute prerequisite to OI treatment in female patients, especially when they claim to not be sexually active.

## Conclusion

The purpose of this study was to describe acne treatment in the IDF in the years 2002–2007. This study provides a unique perspective of acne treatment in a military setting, overall displaying good adherence to general guidelines. The common prescription of oral retinoids to young females without concomitant contraception is alarming.

### Limitations of this study

The data analyzed in this study were collected from the CPR, which was widely in use by 2002, and provided full coverage by 2004. While it is possible that some of the increase in trend was artifactual due to the increase in CPR coverage, this contribution was likely minor and certainly could not account for the continuing rise observed from 2004 and onwards.

## Appendix

**Table 6:** Acne medications, as defined in the CPR and pharmacy management programs (contains only medications located in the database search)

Type	Medication name	Active ingredient/s	Admin.
1st	ACNETRIM/AKNE-MYCIN 2 %	Erythromycin	Topical
1st	BENZAMYCIN	Benzoyl peroxide, Erythromycin	Topical
1st	DALAGIS-T	Clindamycin	Topical
1st	NEO-MEDROL 5 %, Lot.	Aluminium chlorohydroxide complex solution	Topical
		Methylprednisolone acetate	
		Neomycin sulphate	
		Sulfur	
1st	PANOXYL	Benzoyl peroxide	Topical
1st	RETAVIT	Tretinoin	Topical
1st	ZINDACLIN	Clindamycin	Topical
2nd	MINOCIN/MINOCYCLINE 50/100 mg	Minocycline	Oral
3rd	ROACCUTANE/CURATANE 10/20 mg.	Isotretinoin	Oral
non distict acne	ANDROCUR 10 mg., Tab.	Cyproterone acetate	Oral
non distict acne	DALACIN-C 150 mg., Cap	Clindamycin	Oral
non distict acne	DOXYLIN 100 mg., Tab.	Doxycicline	Oral
non distict acne	ERYTHRO TEVA 250/500 mg., Tab.	Erythromycin	Oral
non distict acne	TEVACYCLINE 250 mg, CAPS	Tetracycline	Oral
Oral contraceptives	DIANE 35, Tab.	Cyproterone acetate, Ethynil estradiol	Oral
Oral contraceptives	FEMINET Tab.	Desogestrel, Ethynil estradiol	Oral
Oral contraceptives	GYNERA, Tab.	Ethynil estradiol, Gestodene	Oral
Oral contraceptives	HARMONET, Tab.	Ethynil estradiol, Gestodene	Oral
Oral contraceptives	MELIANE, Tab.	Ethynil estradiol, Gestodene	Oral
Oral contraceptives	MERCILON, Tab.	Desogestrel, Ethynil estradiol	Oral
Oral contraceptives	MICRODIOL, Tab.	Desogestrel, Ethynil estradiol	Oral
Oral contraceptives	MINESSE Tab.	Ethynil estradiol, Gestodene	Oral
Oral contraceptives	MINULET, Tab.	Ethynil estradiol, Gestodene	Oral
Oral contraceptives	ORTHO-CYCLEN, Tab.	Ethynil estradiol, Norgestimate	Oral
Oral contraceptives	YASMIN, Tab.	Drospirenone, Ethynil estradiol	Oral
Supportive care	AQUOSUM, Crm. [K]	Aqueous cream	Topical
Supportive care	VASELINE 10G, OINT	white petrolatum jelly	Topical

## Abbreviations

IDF: Israel defense forces
OC: Oral contraceptive
OI: Oral isotretinoin
HDL: High density lipoprotein
LDL: Low density lipoprotein
CPK: Creatine phosphokinase
CPR: Computerized patient record
ICD9: International classification of diseases
